# Gastroesophageal flap valve reflected EGJ morphology and correlated to acid reflux

**DOI:** 10.1186/s12876-017-0693-7

**Published:** 2017-11-22

**Authors:** Chenxi Xie, Yuwen Li, Ning Zhang, Lishou Xiong, Minhu Chen, Yinglian Xiao

**Affiliations:** Department of Gastroenterology, The First Affiliated Hospital of Sun Yat-Sen University, Guangzhou, Guangdong Province 510080 People’s Republic of China

**Keywords:** Esophagogastric junction, Gastroesophageal reflux, Gastroesophageal flap valve, High-resolution manometry

## Abstract

**Objective:**

The anatomy of esophagogastric junction (EGJ) serves as the anti-reflux barrier. The gastroesophageal flap valve (GEFV) is a component of EGJ. The aim of the current study was to assess its correlation with the esophageal acid exposure and the impact on anti-reflux barrier function by using the metrics of EGJ contraction.

**Methods:**

Eighty three patients with typical GERD symptoms were included in the study. Upper endoscopy, high-resolution manometry (HRM) and 24 h multichannel intraluminal impedance-pH (MII-pH) monitoring were performed in all patients. GEFV was determined as four grades during endoscopic examination based on the Hill classification. The esophageal pressure topography (EPT) metrics defined in the updated Chicago Classification were measured by HRM, including integrated relaxation pressure (IRP), EGJ contractile index (EGJ-CI),expiratory EGJ pressure(EGJP-exp) and inspiratory EGJ pressure (EGJP-insp).

**Results:**

The GEFV grade III and IV was more commonly found in patients with esophagitits (*p* < 0.05). The acid exposure time (AET%) and supine AET% were lower in patients with GEFV grade I (*p* < 0.01). There was weak correlation between AET% and GEFV grades (*r* = 0.27, *p* = 0.013). There were more EGJ morphology type III in patients with GEFV grade IV (*p* < 0.05).There were no significant differences on the values of four HRM metrics among the patients with different GEFV grades (*p* > 0.05).

**Conclusion:**

The GEFV grades were associated with acid reflux positively and could be a good reflection of EGJ morphology in HRM. But it had no impact on the four HRM metrics. Our research revealed that GEFV may play an assistant role in the anti-reflux barrier.

## Background

The dysfunction of anti-reflux barrier was a main cause for gastroesophageal reflux disease (GERD). The esophagogastric junction (EGJ) plays the fundamental role for the barrier. The anatomical structures of EGJ are complex, containing lower esophageal sphincter (LES), crural diaphragm (CD), His angle and flap valve.

The gastroesophageal flap valve (GEFV) is a 180-degree musculomucosal fold apposite to the lesser curvature of stomach as viewed with a retroflexed endoscope [1]. It is created by the intraluminal extension of His angle. The GEFV was first described by Thor et al. in 1987 [2], and the grading system was created by Hill et al. in 1996 [1]. Respiration and taking meal can influence GEFV. Decrease of His angle degree during expiration allows the contact to be closer. The proximal fundus becomes more spherical in the transverse plane when the gastric is filled, which may enhance the strength of anti-reflux barrier [3]. The GEFV grade IV is associated with hiatus hernia [1]. Several studies have reported that the GEFV grades were associated with acid reflux and may be helpful to predict the response of proton pump inhibitor (PPI) treatment [4–7]. LES and CD are recognized as the main determinants of EGJ pressure, but the contribution of GEFV in the strength of anti-reflux barrier is still controversial, because the presence of intact GEFV is not enough to exclude the diagnose of GERD [7]. Transoral incisionless fundoplication (TIF) is useful in reinforcing the valve strength. TIF can reduce the distensibility of EGJ, associated with a decreased volume of refluxte [8, 9]. These results suggest that the intact flap valve may play an assistant role in the anti-reflux barrier and reporting the Hill classification on endoscopy would be helpful in identifying GERD patients.

The high-resolution manometry (HRM) is a useful tool in assessing EGJ function. With the advantage of synchronous and fine fidelity on presenting the peristalsis of whole esophagus, HRM can distinguish EGJ more accurately [10, 11]. There are three traditional metrics used to evaluate the EGJ function. Integrated relaxation pressure (IRP) indicates the adequacy of EGJ relaxation after swallowing. EGJP-insp and EGJP-exp. represent the barrier pressure during respiration separately [12, 13]. Recently a new metric, EGJ contractile integral (EGJ-CI) was developed by F.Nicodeme et al. [13]. EGJ-CI integrates the contraction length and amplitude of EGJ, as well as exclude the influence of respiration rate, so it is superior to those above. Several studies have showed that defective EGJ-CI was more frequently found in GERD patients with pathological acid reflux and lower EGJ-CI was associated with better symptom response after antireflux surgery [14, 15]. The EGJ-CI value would be increased after antireflux surgery [16]. These results support that the new metric is useful in evaluating anti-barrier function of GERD patients and revealing the role of defective flap valve on reflux.

In the study, we aimed to assess the association between GEFV grades and acid reflux, and to explore the impact of GEFV on the anti-reflux barrier by using the HRM metrics.

## Methods

### Subjects

Consecutive outpatients who had heartburn and/or regurgitation as their main complaint were enrolled. The patient’s symptom should last for at least 3 months, and more than 2 days in one week. All the patients underwent the endoscopy in our hospital which was performed by the same physician (Chengxi Xie) and reviewed by the other doctor (Yinglian Xiao) to obtain agreement on the GEFV pattern. Patients would be also excluded if they had the following: with previous esophageal or gastrointestinal surgery, peptic ulcer, gastrointestinal tumor, primary or secondary severe esophageal motility disorders, severe cardiac, renal or pulmonary disease.

The protocol was approved by the Ethics Committee of the First Affiliated Hospital of Sun Yat-sen University. Written informed consent was obtained from all individuals before every procedure.

### Upper endoscopy

The presence of erosive esophagitis was determined and graded based on the Los Angeles Classification [17]. GEFV was graded from I through IV prospectively during endoscopic examination based on the Hill classification [1]. A clear picture of GEFV would be taken on endoscopy by a regular endoscopist (Chengxi Xie), and the grade was confirmed if the other author (Yinglian Xiao) agreed. If they were unable to reach agreement (for 8 patients), the other authors would participate, and the grade was confirmed if more than two-thirds authors agreed on this grading.

**Fig. 1:**
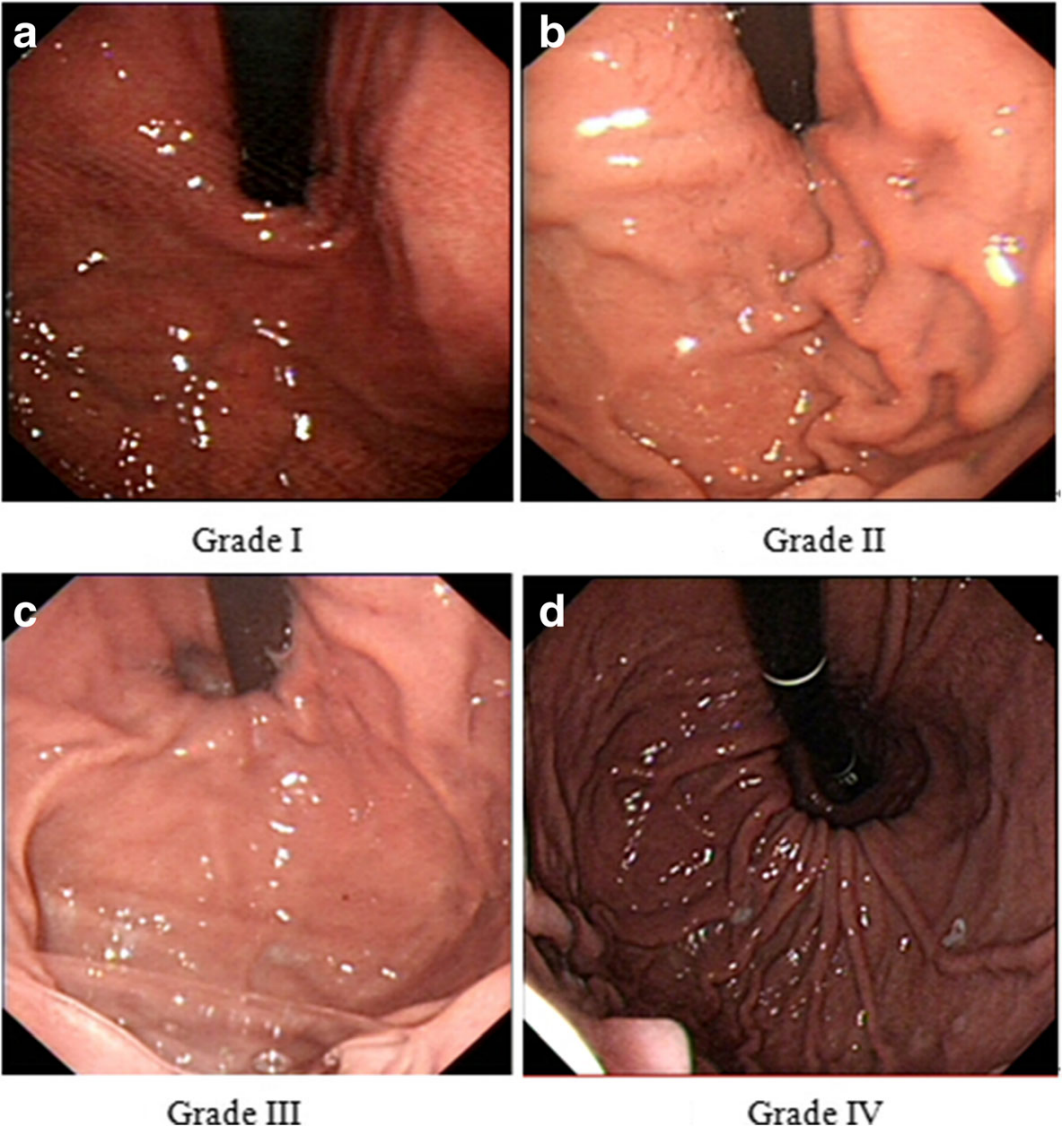
The Hill grades of GEFV. GEFV was graded from I through IV prospectively during endoscopic examination based on the Hill classification: Grade I: The fold of tissue approximated to the endoscope closely; Grade II: The fold of tissue is less prominent than Grade I. The tissue around the endoscope could open during respiration and close rapidly; Grade III: The fold of tissue is barely prominent and the endoscope is not gripped by the tissue tightly; Grade IV: There is no prominent fold of tissue at all and a hiatal hernia is present

The grades of GEFV were shown in Fig. 1.

### High-resolution manometry (HRM)

After fasting for at least 8 h, HRM was performed in all subjects in the supine position. The HRM catheter is assembled with 36 circumferential sensors separated at 1 cm intervals (Given Imaging, Duluth, GA, USA). Transducers were calibrated at 0 and 300 mmHg using externally applied pressure. The catheter was placed transnasally. At least three sensors were placed in the stomach and the changes of pressure could be recorded from upper esophageal sphincter to the stomach. The manometric protocol included a 30s baseline recording and ten 5 mL liquid swallows. The studies were analyzed manually through using the Manoview software (Given Imaging, Duluth, GA, USA).

The EPT metrics were defined by the updated Chicago Classification [18]. The EGJ morphology could be divided into three subtypes according to the relative localization of LES and CD, this was described in the updated Chicago classification [17]. Four metrics were measured in the study, including Integrated relaxation pressure (IRP), EGJ contractile index (EGJ-CI), expiratory EGJ pressure (EGJP-exp) and inspiratory EGJ pressure (EGJP-insp).

**Fig. 2:**
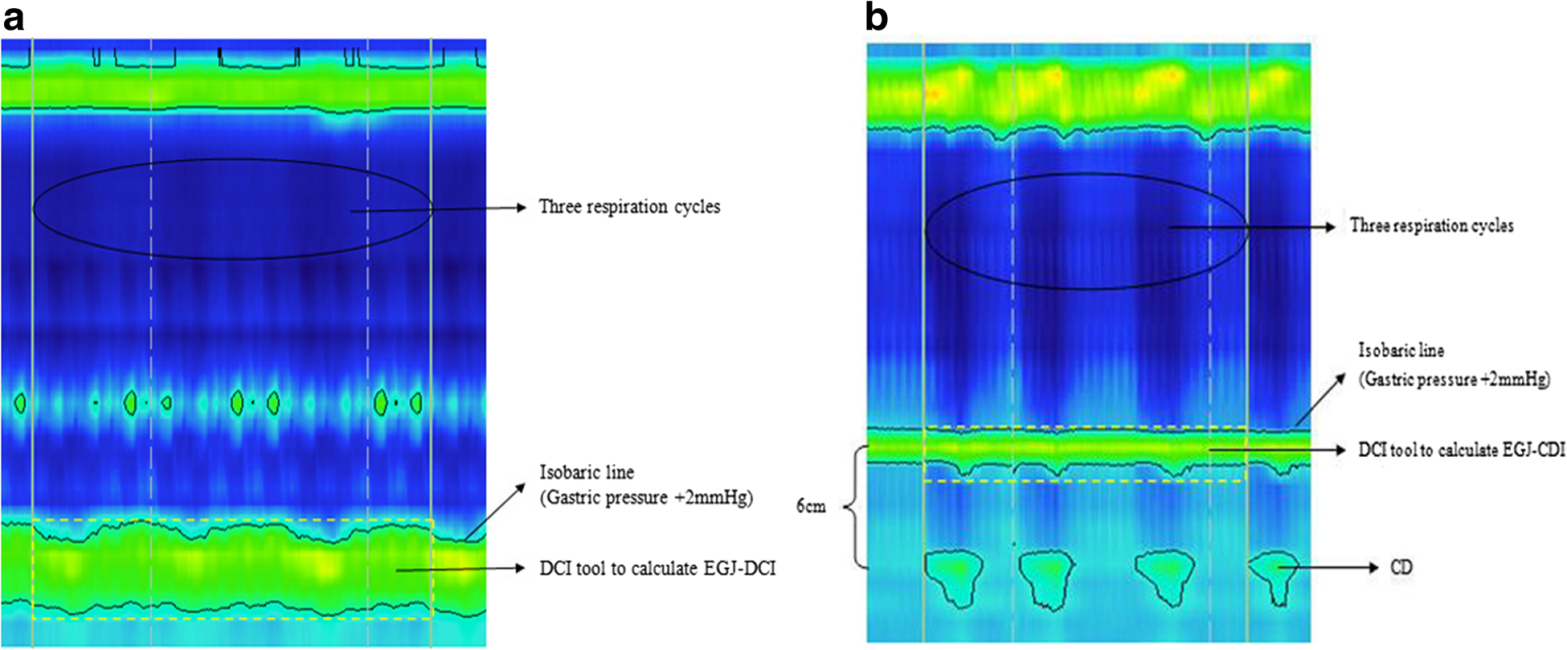
The calculation of EGJ-DCI. At the baseline state, a swallow frame was added on three respiration cycles beginning at the inspiration. Using the isobaric contour, a pressure with 2 mmHg higher than the intragastric pressure was setup as the barrier margin. Then the DCI tool was used to measure the value of EGJ-DCI. **a** The changes of total esophageal pressure at rest were shown by HRM. The yellow box was used to show the region chose to calculate EGJ-DCI. The LES and CD were superimposed. **b** The calculation of EGJ-DCI. The CD component was excluded when the distance between LES and CD was more than 2 cm

It was needed to calculate EGJ-DCI before getting the values of EGJ-CI. The EGJ-DCI was calculated in a quiescent position without swallow. For the patients with EGJ II or III morphology, the LES and CD was separated, the CD component was excluded if the distance between LES and CD was more than 2 cm as shown by HRM [15]. A swallow frame was added on three respiration cycles beginning at the inspiration. Using the isobaric contour, a pressure with 2 mmHg higher than the intragastric pressure was setup as the barrier margin. Then the DCI tool was used to measure the value of EGJ-DCI (Fig. 2). The value then was divided by the duration of the three respiratory cycles to yield EGJ-CI [13].

IRP represented the mean EGJ value of 4 s maximal deglutitive relaxation in the 10s window after the upper esophageal sphincter (UES) relaxation. The median IRP value of 10 liquid swallows were taken into account in the study. EGJP-insp represented the average maximal inspiratory EGJ pressure and EGJP-exp. represented the average EGJ pressure midway between inspirations. They were calculated at the same three respiratory cycles chosen to measure EGJ-DCI.

### Twenty-four-hour multichannel intraluminal impedance-pH (MII–pH) monitoring

All subjects underwent 24 h monitoring by using an ambulatory MII-pH monitoring system (Sleuth; Sandhill Scientific, Inc.; Highland Ranch, CO, USA). The pH electrode was placed at 5 cm above the upper margin of the lower esophageal sphincter (LES), and impedance were recorded at six sites (3, 5, 7, 9, 15, and 17 cm above the LES, respectively).The meal time was excluded from the analysis.

**Fig. 3:**
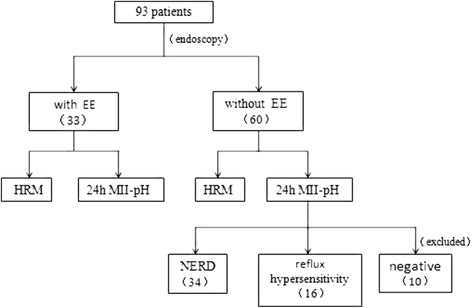
The flow chart of research

The NERD patients were determined as without esophagitis, but with abnormal esophageal acid exposure (acid exposure time(AET) ≥ 4%). And the patients with reflux hypersensitivity was determined as without esophagitis and pathological acid reflux, but with symptom association probability ≥ 95%. Patients with heartburn for more than 2 days a week, without apparent correlation to acid or nonacid reflux and with poor response to optimized PPI treatment should be classified as with “functional heartburn (FH)” [19, 20]. In the study, the patients without EE and positive 24 h MII-pH monitoring results were excluded, because only these manifestations were not sufficient to make the diagnosis of FH. A flow chart was shown in Fig. 3.

### Statistical analysis

The one-way ANOVA was used to compare the differences if the data followed normal distribution. Rank sum test was used if the data were presented as median (interquatile range).The spearman rank correlation was used to explore the correlation between AET% and the GEFV grades and the correlation between supine AET% and EGJ morphology. The *p* value less than 0.05 were considered statistically significant. Statistical analysis was completed by using SPSS 20.0 (SPSS Inc., Chicago, IL, USA).

## Results

### Demographic characteristics of the patients

Thirty-three patients with erosive esophagitis (7 patients with LA-A grade, 24 patients with LA-B grade, 2 patients with LA-D grade), 34 patients with NERD and 16 patients with reflux hypersensitivity were included in the study (Table 1). Ten patients without EE and positive 24 h MII-pH monitoring results were excluded. For all the patients, 7 with GEFV grade I, 47 with GEFV grade II, 20 with GEFV grade III and 9 with GEFV grade IV. A hiatal hernia was present when the GEFV IV was found under endoscopy.

**Table 1:** Demographic data of the patients

	EE (*n* = 33)	NERD (*n* = 34)	Reflux Hypersensitivity (*n* = 16)
BMI(kg/m^2^)	24.09 ± 2.85	23.89 ± 4.04	23.15 ± 2.73
Male	24	18	6
Age(years)	51.64 ± 15.03	45.18 ± 11.53	49.19 ± 12.75

51.5% patients with GEFV III/IV were included in the EE group, 26.5% in the NERD group and 18.8% in the group with reflux hypersensitivity (*p* = 0.032, Table 2).

**Table 2:** The different distribution of GEFV grades between patients with and without erosive esophagitis

	GEFV I/II	GEFV III/IV	*p* value
EE	16	17	0.032
NERD	25	9	
Reflux Hypersensitivity	13	3	

### The correlation between GEFV grades and the esophageal acid exposure

The patients with GEFV grade I had lower AET% and supine AET% than other groups (*p* < 0.01), but the difference among groups with GEFV grade II to grade IV was not significant. Though there was an ascending trend on the upright AET% when the GEFV grades increased, the differences were not significant when compared between any two groups separately (*p* > 0.05). There was no significant difference on the reflux episodes within four groups (*p* > 0.05) (Table 3).

**Table 3:** GEFV grades associated with esophageal acid exposure

	GEFV I (*n* = 7)	GEFV II (*n* = 47)	GEFV III (*n* = 20)	GEFV IV (*n* = 9)	*p* value
AET%	0.6(0.1, 3.6)	5.2(2.9,8.4)^a^	6.6(3.4,11.4)^a^	7.7(2.4,22.4)^a^	0.019
Supine AET%	0(0, 0.1)	0.8(0,4.5)^a^	0.6(0,4.7)^a^	5.0(1.1,9.1)^a^	0.009
Upright AET%	1.2 (0.2,6.6)	7.1(3.9,12.6)	9.4(3.3,16.5)	11.1(3.8,33.2)	0.049
Total reflux episodes	37.0(18.0,70.0)	54.0(38.0,69.0)	56.50(35.0,74.8)	62.0(48.0,74.5)	0.639
Supine reflux episodes	3.0(0,22.0)	5.0(2.0,12.0)	4.5(1.0,7.8)	10(7.0,25.0)	0.109
Upright reflux episodes	36.0 ± 20.6	47.0 ± 24.1	50.1 ± 27.8	44.0 ± 22.4	0.617
EGJ-exp.(mmHg)	15(9,28)	10(4,15)	8(5,15.8)	4(2,16.5)	0.418
EGJP-insp(mmHg)	23(13,36)	16(12,23)	16.50(11.25,21.50)	18(6,24)	0.338
EGJ-CI(mmHg.cm)	31.5 (27.0,50.4)	25.6 (17.9,34.6)	27.3 (17.3,37.3)	20.0 (11.8,22.5)	0.193
Median IRP(mmHg)	12(9.3,13.2)	8.5(5.9,12.4)	8.9(6.5,14.2)	8.8(5.2,10.0)	0.160

Most parameters were presented as medians (interquartile range) except the upright reflux episodes

^a^different from the GEFV I group

**Fig. 4:**
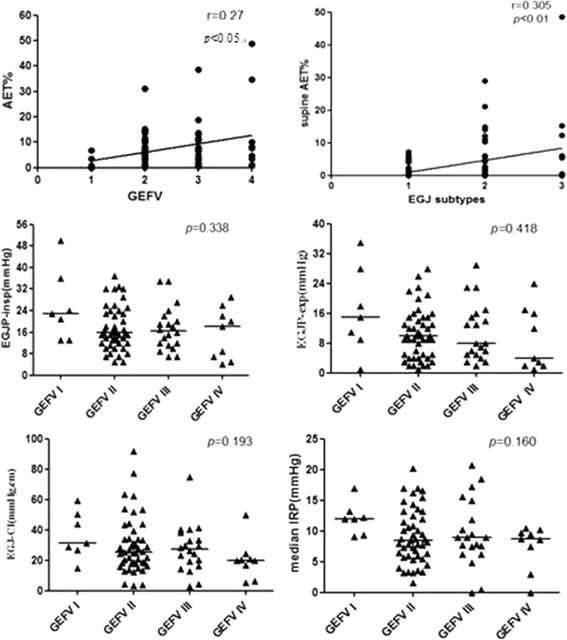
GEFV grades correlated to AET% weakly, but had no impact on the values of four HRM metrics. EGJ subtypes correlated to supine AET% positively. The spearman rank correlation was used to explore the correlation between AET% and the GEFV grades and the correlation between supine AET% and EGJ morphology

There was a weak correlation between GEFV grades and the AET% (*r* = 0.27, *p* = 0.013). The EGJ morphology was correlated to supine AET% positively (*r* = 0.305, *p* = 0.005). (Fig. 4).

### Comparison of HRM findings among patients with different GEFV grades

The distribution of EGJ subtypes in patients with different GEFV grades were shown in Table 4. Because GEFV IV and EGJ III was associated with hernia, they were listed separately. The EGJ subtype III was more common to be found in patients with GEFV IV than that without (55.56% vs 6.76%, *p* < 0.05). Though the values of four EPT metrics seemed higher in patients with GEFV grade I, there were no significant differences among the four groups (*p* > 0.05) (Fig. 4, Table 3).

**Table 4:** The distribution of EGJ subtypes in patients with different GEFV grades

	EGJ subtype I/II	EGJ subtype III	*p* value
GEFV I-III	69	5	0.00
GEFV IV	4	5	

Though there was a descending trend on EGJ-CI in patients with GEFV IV, the difference was not significant when compared to that without (20.03(11.76, 22.49) mmHg.cm vs 26.82(18.27, 38.69) mmHg.cm, *p* = 0.092).

There were no correlations between GEFV grades and the HRM metrics (*p* > 0.05).

## Discussion

The dysfunction of EGJ implied the abnormal flow of gastric content into the esophagus. The LES and crural diaphragm are recognized as the two key components of the anti-reflux barrier [21]. GEFV is a special musculomucosal fold reflecting the contact of proximal gastric fundus with the esophageal cavity. The connection between proximal stomach and the esophagus would be closer when the GEFV grade decreased [1]. It seems that GEFV may play a synergistic role in the anti-reflux barrier, but there is still lack of sufficient research. HRM has the advantage to better identify the anatomic structure of EGJ and could display the barrier as the distal high pressure zone (DHPZ) [22]. EGJ-CI is a new HRM metric, which was first calculated by Nicodeme et al. [13], and they found that in patients with refractory GERD, its value was lower only in the group fulfilled all the criteria of GERD. It seems that the metric is useful in evaluating the contraction of EGJ at rest. So we assessed the correlation between GEFV grades and the acid reflux in the study and used EGJ-CI and other traditional HRM metrics to explore the impact of GEFV on the anti-reflux barrier function. It turned out that the grades of flap valve was associated with esophageal acid exposure weakly, and the differences on EGJ-CI were not significant within patients with different GEFV grades.

The increasing separation between LES and CD can cause an increase in AET% and reflux episodes [23]. EGJ subtype III means that the separation of CD and LES peaks is >2 cm. It is more common to be found in patient with higher GEFV grade. This suggested that the grade of GEFV was indeed a reflection of anti-reflux barrier. Contractor et al. showed that the GEFV grades were correlated with the severity of esophagitis positively [24], and Chang et al. reported that the grade I and II was less to be found in EE patients [25]. This was in line with our results. In fact, the grades I and II of GEFV were commonly found in healthy controls [1]. This result suggested that the dysfunction of GEFV may be benefit for pathological acid reflux. Because grade I implies that the fold of tissue along the lesser curvature would grip the endoscope closely, it was not surprising to find that the esophageal acid exposure in this group was not severe. The difference on AET% between patients with GEFV I and that without was more significant when the patients were in supine position. This may due to the lack of gravity effect in this position, thus the anti-reflux barrier became more important in the protection from regurgitation. EGJ subtypes were correlated to supine AET%. This result supported that GEFV could be the reflection of EGJ morphology. The GEFV grade IV is known to be associated with hiatus hernia [1], so it was reasonable to find that the AET% values in this group seemed higher than the others no matter in supine or upright position. The GEFV grade II and III also indicated a defect on anti-reflux barrier, so there was an ascending trend on the AET% and reflux episodes. Routinely reporting the impaired GEFV grades on endoscopy would be helpful in finding out the potential patients with excessive esophageal acid exposure.

Koch et al. showed that the flap valve had no significant effect on the LES pressure [7]. EGJ-CI was a comprehensive reflection of the EGJ contraction at rest. It captures more factors attributing to the barrier function. Tolone S et al. found that patients with defective EGJ-CI may have positive 24 h MII-pH monitoring more frequently [26]. We had found that EGJ-CI was correlated with esophageal acid exposure in the supine position [27]. These results supported that calculating EGJ-CI was useful in evaluating the defect of barrier function. In the study, though the impaired GEFV benefitted acid reflux in the same position and the values of EGJ-CI seemed lower when the grades increased, there were not significant differences within the four groups. We also found that the EPT metrics reflecting EGJ contraction during respiration were similar within different GEFV groups. These results suggested that the GEFV function was not the determining factor of the barrier function, though the patients with lower EGJ-CI and higher GEFV grades may be benefit from the antireflux surgery [14–16]. A reasonable interpretation was that the role of GEFV was not as important as that of LES and crural diaphragm in the strength of anti-reflux barrier. In fact, the barrier strength is not a simple sum of the effect of all the structures located in EGJ, the LES tone may hinder the transmission of extrinsic pressure [21]. This was supported by the result that no correlation was found between GEFV grades and the HRM metrics reflected EGJ contractility. This may explain why the correlation between AET% and GEFV grades was weak and the difference on AET% in patients with impaired GEFV was not significant. Further research on the function of GEFV demands measurements using technique with higher sensitivity in differentiating EGJ pressure components, such as 3D–HRM technology.

There were two limitations in our study. One was that the mucosal erosion of most EE patients was not severe. If more patients with LA-C or LA-D grades were enrolled in the study, more patients with higher GEFV grades could be included. This would be useful in acquiring a greater depth on the correlation between impaired GEFV grades and acid reflux. The other limitation was that we did not included “functional heartburn (FH)” patients in the study. The comparison of the distribution of GEFV grades between GERD and FH patients would be useful in further revealing the valve function on the protection from regurgitation.

## Conclusions

In summary, our findings in the study suggested that the GEFV grades had weak impact on the esophageal acid exposure, and it was a good reflection of EGJ morphology in HRM. The valve may play an assistant role on the strength of anti-reflux barrier and was needed to report on endoscopy. Further research on the therapeutic value of impaired valve was still needed for the long-term management of GERD patients.

## Abbreviations

AET%: acid exposure time
CD: crural diaphragm
DHPZ: distal high pressure zone
EE: erosive esophagitis
EGJ: esophagogastric junction
EGJ-CI: EGJ contractile index
EGJP-exp: expiratory EGJ pressure
EGJP-insp: inspiratory EGJ pressure
EPT: esophageal pressure topography
FH: functional heartburn
FH: functional heartburn
GEFV: gastroesophageal flap valve
GERD: gastroesophageal reflux disease
HRM: high-resolution manometry
IRP: integrated relaxation pressure
LES: lower esophageal sphincter
MII-pH: multichannel intraluminal impedance-pH
NERD: nonerosive reflux disease
PPI: proton pump inhibitor
UES: upper esophageal sphincter
